# Primary school characteristics in Sokoto State, Nigeria

**DOI:** 10.1038/s41597-026-07167-6

**Published:** 2026-04-03

**Authors:** Lisa Bogler, Sophie Ochmann, Kehinde Elijah Owolabi, Ann-Charline Weber, James Olaniyi Okunlola, Sebastian Vollmer

**Affiliations:** 1 Centre for Modern Indian Studies and Department of Economics, University of Goettingen, Göttingen, Germany; 2 Department of Agricultural Extension and Communication Technology, Federal University of Technology, Akure, Nigeria

**Keywords:** Education, Developing world

## Abstract

This study gathered evidence on the state of primary schools in Sokoto State, Nigeria, in 2018 and 2019. The datasets contain two survey waves of a sample of 128 primary schools. Data was collected during unannounced visits to each school using several survey tools, including an observational survey of the school, interviews with the headmaster, teachers, and school-based management committee members. Topics covered in the interviews include school characteristics, the financial situation of the school, cooperation between stakeholders, the role of the school-based management committee, teaching methods, and absenteeism. While the data was primarily collected for the purpose of the evaluation of a school development programme, it can also be used to understand the state of primary schools in Sokoto, Nigeria.

## Background & Summary

The importance of primary education has been widely recognized^1,2^. To measure progress towards achieving universal primary school attendance as well as learning, recent and high-quality data are needed. However, data on the state of schools from poor and fragile contexts remain scarce and of low quality^3^. This data contributes towards filling this evidence gap on the state of primary school education in low-resource and fragile settings.

This data was collected during a randomized controlled trial for the evaluation of the school development programme *Nigerian Partnership for Education Project* (NIPEP)^4^. Two waves of data were collected, in July and August 2018 before programme rollout and in November and December 2019 after programme rollout. In a sample of 128 primary schools, several survey tools were implemented during unannounced visits to the schools: an observational survey of the school and interviews with the headmaster, teachers, and members of the school-based management committee (SBMC). The observational survey captured activities going on at the school at the moment of the visit, information copied from school registries, and characteristics of school buildings and compound. Interviews with headmasters, teachers, and SBMC members captured personal characteristics of the respondents, further school characteristics, opinions on absenteeism among teachers and pupils, opinions on differential treatment of boys and girls, ongoing and past interventions from which the school benefitted, the financial situation of the school, and cooperation between stakeholders.

This data can be used to further explore the state of primary schools in Sokoto State, Nigeria, including the functioning of SBMCs. The observational tool for unannounced visits can be implemented elsewhere using the published survey tool.

## Methods

### Ethical considerations

The evaluation study was conducted in cooperation with the Nigerian Federal Ministry of Education and received ethical clearance from the National Institute of Social and Economic Research, Ibadan, Nigeria (NISER/RMD/19/05). Informed consent was taken from respondents orally prior to interviews with headmasters, teachers, and SBMC members. The respondent specifically consented to participation in the interview and the release of the data for Nigerian and international researchers in an anonymous, de-identified form. While the original study protocol planned for written informed consent, the survey firm that was hired to collect the survey data deviated from this with the permission of the Nigerian Federal Ministry of Education, without prior agreement from the research team. This deviation was accepted given that all adults were interviewed in their function related to school (teacher, headmaster, SBMC member) and the Ministry agreed to the procedure, confirming that verbal consent was standard practice in school based public surveys in Nigeria.

### Study setting and design

The study was conducted in the state of Sokoto in North-West Nigeria on the border with Niger. It is one of the poorest states in Nigeria. According to the Demographic and Health Survey 2018, 52 percent of households belong to the lowest wealth quintile of Nigeria^5^. Sokoto’s high poverty incidence is also expressed in the small rate of 34.4 percent of households having an improved source of drinking water and an under-5 mortality rate of 197 deaths per 1000 live births compared to 132 in Nigeria as a whole. 54.8 percent of children were stunted, an indication for cumulative growth deficits due to poor nutrition. The primary school net attendance ratio was 28.9 percent^5^.

The data was collected as part of the NIPEP impact evaluation, in a baseline in 2018 and an endline in 2019. NIPEP, a large-scale programme to improve access to and quality of basic education, was implemented between 2015 and 2019 in five north western states by federal and state ministries and education boards. Between 2018 and 2019, select components of NIPEP were evaluated in Sokoto using a randomised controlled trial in primary schools in nine of the 23 districts, called Local Government Areas (LGAs), of Sokoto State. The evaluation focused on school improvement grants and the training for SBMC members. Results of the evaluation are presented elsewhere^4^. The data described here originates from the data collection for the randomized controlled trial evaluating NIPEP.

The selection of schools for the sample was driven by the overall NIPEP programme and logistical considerations. As the evaluation took place in the last implementation phase of the overall NIPEP programme, the sample was limited to schools which met eligibility criteria for NIPEP at the time of the evaluation but had not yet been included in the project in earlier implementation phases. These schools that had not been included in earlier implementation phases because they had not been able to meet eligibility criteria before, such as setting up a bank account and an active SBMC and only fulfilled these criteria at the time of the evaluation. These schools were therefore likely less functional and had less engaged SBMCs than other schools in the same area that had been eligible earlier, but they were also not among the least functional schools that never met eligibility criteria. The research team in Göttingen received a list of schools that were eligible at the time of the evaluation from the NIPEP implementation team and added further selection criteria. Initially, the list contained 777 schools that were eligible for but had not yet received the NIPEP programme. Schools that received programmes similar to NIPEP had to be excluded from the trial, leaving 352 schools. Following the advice of a local partner, schools in very distant and hard-to-reach local government areas were excluded for safety reasons, reducing the sample by 129 schools.

Schools with no grade 2, or with fewer than 35 or more than 160 children enrolled in grade 2 according to official school registers were also excluded due to the evaluation’s focus on grade 2. This process resulted in a sample of 128 schools for the randomised controlled trial and therefore the assignment to treatment or control group and data collection.

### Questionnaire design and content

The research team in Göttingen designed the questionnaires in cooperation with the research team in Nigeria and piloted them in Sokoto. The survey tools were created to capture the outcomes and control variables that were relevant for the NIPEP impact evaluation and conceptual constructs were not used during their design. Supplementary Table S.1 maps all available variables to broader subject domains.

The following topics were covered:

Observational survey:

- Activities ongoing at the school at the moment of the unannounced visit
- Number of teachers employed, absenteeism
- Number of pupils registered, absenteeism
- Timetables of grades 2 and 3 (at baseline only)
- Characteristics of the school buildings and compound

Interviews with headmaster, teachers, SBMC members:

- Further school characteristics
- Opinions on absenteeism among teachers and pupils
- Opinions on differential treatment of boys and girls
- Ongoing and past interventions from which the school benefitted
- Financial situation of the school
- Cooperation between stakeholders (headmaster, teachers, SBMC, parents)
- Roster of all SBMC members capturing sociodemographic information and literacy

The observational survey aimed to capture regular school activities upon arrival at the school before routines were interrupted by the data collection. It started with an enumerator quickly moving from classroom to classroom, capturing whether a teacher was present and the availability of material in each room. The second part was a more detailed observation of specific classrooms to capture activities of teachers and students. Given the state of schools observed at baseline, this more detailed observation of activities in classrooms was dropped from the endline survey. For both parts, it was essential that the visit to the school was unannounced. The remaining modules of the observational survey copied information from registries and timetables and captured observable school characteristics that could be completed with the support of school staff.

All questionnaires were translated to Hausa, the local language, by research assistants in Sokoto.

### Data collection and sample selection within schools

Data collection followed the same process at baseline and endline. A Nigerian survey firm hired enumerators who were fluent in the local dialect of Hausa. All enumerators were trained in the survey tools and used tablets to collect the data. A team of research assistants accompanied the training and monitored data collection.

Data collection was supposed to be conducted during school hours. One enumerator was to collect the observational survey data of the school upon arrival at the school. The remaining enumerators would conduct surveys with the school staff. Among the SBMC members, the chair, vice chair, treasurer, women leader, pupil representative, and one additional available member were supposed to be interviewed. Among teachers, all teaching staff including volunteers and interns of grades 2 and 3 were supposed to be interviewed.

There were several deviations from the data collection plan as schools were often found to be closed at the time of enumerators’ visits, with no adult or child present. At baseline, the observational survey started with recording activities in each classroom, and no teacher was found present in any class at the time of the visit in 63% of schools. Given the experience at baseline, the observational survey was adjusted for the endline to capture the situation at the time of the visit more rapidly and accurately. At endline, no learning activity was going on in 77% of schools, no teacher was present in 46%, no headmaster in 56%, and no pupil in 32% of schools. Overall, 29% of schools were found with no person (adult or child) present at endline. If a school was found closed at the time of the visit, enumerators would ask adults present around the school about the whereabouts of teachers, headmaster, or SBMC members. If any targeted respondent was found and could come to the school, they were interviewed. This procedure resulted in 59 schools (46%) with all survey types completed at baseline and 64 schools (50%) at endline. Unfortunately, the data does not capture whether a particular respondent was present at the school at the time of the visit or was called to the school for the purpose of the interview. For 6 schools at baseline and 3 schools at endline, no survey with any adult could be completed, while the school observation was completed for all sampled schools at baseline. Schools closed at the initial visit were not replaced by others from the sampling frame as the survey sample included all schools that met the survey inclusion criteria. All schools visited at baseline were re-visited at endline. Table 1 gives an overview of records per wave and survey tool. Headmasters only completed the headmaster survey, even if they also acted as teacher at their school.

**Table 1:** Overview of records per wave and survey tool.

	Baseline (2018)	Endline (2019)
School observation	128 (100%)	125 (98%)
Headmaster survey	88 (69%)	99 (77%)
Teacher survey	181 from 81 schools (63%)	175 from 80 schools (63%)
SBMC member survey	285 from 111 schools (87%)	348 from 116 schools (91%)
All survey types	59 (45%)	64 (50%)

Note: Absolute number of observations per survey tool and wave, with percent of schools covered by survey tool and wave out of total sample of 128 schools in parentheses.

Considering baseline and endline, 95 out of 128 schools (74%) have at least one of each survey type (school observation, headmaster survey, teacher survey, SBMC member survey) completed, while the remaining 33 schools miss at least one of the survey types. 12 schools (9%) have no headmaster survey in either baseline or endline, 24 (19%) have no teacher survey, but only 3 have no headmaster and no teacher survey. Only 1 school has no SBMC member survey completed in either baseline or endline. The high number of schools with missing data could raise concerns about representativeness even within the eligible sample. Table 2 compares some basic characteristics (official number of pupils in grade 2 and official number of teachers according to administrative records, share with toilets and number of rooms according to school observation at baseline) for schools with different surveys completed.

**Table 2:** School characteristics.

	Number of schools	Number of pupils in grade 2 (median)	Number of teachers (median)	Share with toilets (%)	Number of rooms (median)
Each type completed	95	55	4	25.3	3
Any type missing	33	54	3	12.1	1.5
No headmaster survey completed	12	48	3	25.0	1
Any headmaster survey completed	116	55.5	4	21.6	3
No teacher survey completed	24	57	3	8.3	2
Any teacher survey completed	104	55	4	25.0	3
No headmaster and no teacher survey completed	3	45	3	33.3	2.5
Any headmaster or teacher survey completed	125	55	4	21.6	3
No SBMC member survey completed	1	—	—	—	—
Any SBMC member survey completed	127	55	4	22.0	3

Note: Absolute number and basic characteristics of schools with different survey tools completed in either baseline or endline, median number of pupils in grade 2 according to administrative school records, median number of teachers according to administrative school records, share of schools with toilets observed during baseline, median number of rooms observed at baseline. No information for category with single observation due to identification risk.

## Data Records

Data for this study is available at the University of Goettingen data depository platform, Göttingen Research Online^6^, with this section being the primary source of information on the availability and content of the data being described. The data folder comprises the following files: the baseline and endline data of the four survey tools, the questionnaires at baseline and endline in English, and a codebook. The data files are in MS Excel format. The questionnaires are in PDF format. The codebook is in MS Excel format and contains a separate tab for each survey tool and wave. Each tab has three columns: (1) “Variable Name” which corresponds to its associated data column in the data files, (2) “Question” which corresponds to the survey question, and (3) “Answer options” which presents the response option categories and their codes.

The data has undergone basic cleaning. This manipulation included cleaning of school identification data and codes for enumerators and supervisors, and renaming of variables for readability and matching between waves. The data has been anonymised by dropping location information and names and by manipulating school identification data.

We added administrative information on school level, which was collected prior to data collection for the purpose of sample selection, to each data file. This information includes school location (rural/urban), school type (regular/islamiyya/nomadic), whether the school was beneficiary of any intervention other than NIPEP, number of children enrolled in grade 2, and the number of teachers employed. We also added information on treatment assignment, namely a dummy on whether the school was assigned to the control group or any treatment group in the randomized controlled trial, and a variable differentiating between the two treatment groups. As NIPEP did not achieve measurable impact^4^, the treatment indicators may not be relevant for many analyses but are included for completeness and transparency. A binary variable indicating that the observation was collected during endline was added to each data file of the second wave.

## Technical Validation

We piloted each survey tool prior to the launch of the baseline survey to ensure the accuracy of the tools and their clarity to respondents. The research team, supported by research assistants, monitored the data collection. However, several aspects during data collection did not go as planned and high data quality could not be ensured. However, several measures can be taken to check validity in data analysis: Enumerator identification codes are included to allow for enumerator controls. As several questions were posed to different respondents, information can be checked by comparing responses between survey tools. While we note that there are some issues with data quality, we believe that it is the best data available in this context.

## Usage Notes

Survey tools can be matched using the manipulated school identification information (school_code), which is included in each data file. Variable names were adjusted to allow for easy appending of the two waves for each survey tool. It was not feasible to track and match teachers or SBMC members across waves, so the data on individual level should be considered independently pooled cross sections. Using school identification information, the data can further be matched with data of a learning assessment conducted with school-aged children in the same setting. This learning assessment is analysed and described elsewhere^7,8^.

Access to the data files of baseline and endline data is controlled as the small number of schools, together with information about school type and size, could risk the identification of schools, and consequently of their headmasters, teachers, and SBMC members. Potential users need to apply for access by signing the data usage agreement available in the data repository, submitting it to the contact provided in the data repository (click on “contact owner”), and following the process in the repository for requesting the files. Users’ identities will be validated, and their names, institutions and purpose of data use will be recorded. While a statement about the purpose of data use is requested to collect data use cases, no screening on project topic will be conducted. Applications will generally be responded to within two weeks.

## Supplementary information

Supplementary Material

## Data Availability

Data for this study is available at the University of Goettingen data depository platform, Göttingen Research Online^6^.

## Code availability

No custom code was used to generate this study.
