# Supplementary material for: Primary school characteristics in Sokoto State, Nigeria
Source: Sci Data. 2026 Apr 3;13:539. doi: 10.1038/s41597-026-07167-6 (PMC13056961; doi:10.1038/s41597-026-07167-6)
Supplement: Supplementary file 1 — Supplementary Material [file 41597_2026_7167_MOESM1_ESM.pdf]

Primary school characteristics in Sokoto State, Nigeria

Lisa Bogler, Sophie Ochmann, Kehinde Elijah Owolabi, Ann-Charline Weber, Niyi Okunlola, Sebastian Vollmer

Supplementary Material

Table S.1. Subject domains and variables in each survey tool

Topic domains	BL Headmaster	EL Headmaster	BL SBMC	EL SBMC	BL Teachers	EL Teachers	BL Observations	EL Observations
**Teacher and pupil absenteeism**	HOT03-HOT04, HOT09, HOP05	HOT03-HOT04, HOP05	SCSQ04, SCSQ09		TTA01-TTA04, TVE05	TTA01-TTA04	LG02*, Snap*_OBS03, Snap*_OBS11-Snap*_OBS13, TD03-TD04, SD03a*-SD03c*	LG02* OBSA01-OBSA03 TD03-TD04, SD03a*-SD03c*
**Teacher and pupil activity**	HPC13, HPC22-HPC24, HSC13-HSC14, HOT05-HOT08	HPC13, HPC22-HPC24, HSC13-HSC14, HOT05-HOT08	SCPC16		TPC18-TPC24, TPT01-TPT02, TTPS01-TTPS16, TTPS19-TTPS25, TTPG03-TTPG06, TPS10, TVE06	TPC18-TPC20, TPC23-TPC24, TPT01-TPT04, TTPS01-TTPS04, TTPS08-TTPS25, TTPG03-TTPG06, TPS10	Snap*_OBS01-Snap*_OBS02, Snap*_OBS04-Snap*_OBS08, Classes*M-Classes*F	LG00
**Respondent's characteristics and attitudes**	HPC09-HPC14, HPC21, HOP01, HOP04, HPC02, HPC04-HPC07, HSC15-HSC18, numP2	HPC09-HPC14, HPC21, HPC02, HPC04-HPC06, numP2	SCPC06-SCPC13, SCPC16, SCPC18, SCSQ03, SCPS02-SCPC05, SCMC03_*-SCMC10_*, numP2	SCPC06-SCPC13, SCPC16, SCPC18, SCPS02-SCPC05, SCMC04_*-SCMC10_*, numP2	TPC09-TPC12, TPS01-TPS02, TPS08-TPS13, TTPG01-TTPG02, TPG02-TPG12, TVE02-TVE04, TVE07-TVE09, TVE12-TVE13, TVE14, TPC02-TPC04, TPC06-TPC08, TTPG01-TTPG02, TVE14c-TVE14e, numP2	TPC09-TPC12, TPS01, TTPG01-TTPG02, TPG03, TPG05-TPG08, TPG10-TPG12, TVE07-TVE09, TVE12-TVE13, TVE14, TPC02-TPC04, TPC06-TPC07, numP2	TD02a-TD02b, SD01*-SD02*, SD04*-SD06*, numP2	SD01*-SD02*, SD04*, numP2

**Gender equity**	HOP02-HOP03, HOP08-HOP09, HSP08	HOP02-HOP03, HOP08-HOP09, HSP08	SCF10, SCW01-SCW11	SCF10, SCW01-SCW02, SCW05-SCW07, SCW11	TVE07-TVE08, TVE12-TVE13	TVE07-TVE08, TVE12-TVE13		
**Resource availability and school equipment**	HSC09-HSC12, HOT02, HCO18-HCO19	HSC09-HSC10, HSC12, HOT02, HCO18	SCSQ05-SCSQ08	SCSQ05-SCSQ06	TPS03- TPS07, TPS14, TTPS01-TTPS03, TTPG07	TPS03, TPS05-TPS07, TPS14, TTPS01-TTPS03, TTPG07	LG05*-LG11*, Snap*_OBS09-Snap*_OBS10, Snap*_OBS14, SC01-SC41	LG05*-LG11*
**School finanacial practices**	HPC15-HPC20, HSP09-HSP13, HFS01-HFS11	HPC17, HSP09-HSP13, HFS06-HFS08, HFS11	SCPC14-SCPC15, SCSB01-SCSB05, SCSB11-SCSB12, SCFS01-SCF09, SCSP01-SCSP03, SCSP07-SCSP09	SCSB04, SCSB11, SCFS03, SCSP01-SCSP03, SCSP13-SCSP17	TPC13-TPC17	TPC13, TPC16		
**Community participation**	HCO16, HSP12, HSF07-HSF08, HSIP02, HCO01-HCO15	HSP12, HSF07-HSF08, HSIP02, HCO01-HCO02, HCO06, HCO08, HCO10	SCF01-SCF03, SCF12-SCF17, SCFS03-SCFS04, SCF18, SCP01-SCP03, SCP05-SCP07, SCP09, SCCO01-SCCO03, SCSP04, SCSP06, SCT01-SCT05, SCSP06, SCSP10, SCSP12, SCMC01-SCMC02, SCSB12, Roster	SCF01-SCF02, SCF12-SCF17, SCFS03, SCF18, SCP07, SCT01-SCT05, SCSP04, SCSP06, SCSP16, SCMC01-SCMC02, Roster	TVE14, TCO10-TCO15	TVE14, TCO10-TCO12, TCO14		

**School governance**	HPC23-HPC27, HSC01-HSC08, HSC13-HSC20, HSC19-HSC20, HOT01, HOT05-HOT08, HSP01-HSP07, HSIP01-HSIP02, HSC07-HSC08, HCO10-HCO17, type, num_teacher	HPC23-HPC27, HSC01-HSC07, HSC13, HSC19-HSC20, HOT01, HOT05-HOT08, HSP01-HSP04, HSIP01-HSIP02, HCO10, type, num_teacher	SCF01-SCF04, SCF06-SCF11, SCW01-SCW11, SCPU01-SCPU05, SCF18, SCP01-SCP03, SCP05-SCP07, SCSB11, SCSQ01-SCSQ02, SCT01-SCT05, SCP06b, SCP06, SCF04b, type, num_teacher	SCF01-SCF02, SCF04, SCF06-SCF10, SCW01-SCW02, SCW11, SCPU01-SCPU02, SCSB11, SCF18, SCP06-SCP07, SCSQ01, SCT01-SCT05, SCSP16, SCP06b, SCF04b, type, num_teacher	TPT01-TPT02, TPS01-TPS02, TPS08-TPS13, TPS15-TPS16, TCO01-TCO09, TTPS23-TTPS25, type, num_teacher	TPT01-TPT04, TPS01, TPS15-TPS16, TTPS23-TTPS25, TCO02, TCO05-TCO07, type, num_teacher	No_Of_Class, TD01a-TD01h, TD03, type, num_teacher	No_Of_Class, TD00A, TD00B, TD01a-TD01h, TD03, SD0001, SD0002, type, num_teacher
**Internal project variables**	DateofInterview, StartTime, school_code, LGA_code, Respondentdesignation, Respondentdesignation_other, consent, EndTime, interviewer_code, supervisor_code, rural, type, oth_interv, numP2, num_teacher, treattype, treatment, treatdummy, endline_dummy	DateofInterview, StartTime, school_code, LGA_code, Respondentdesignation, Respondentdesignation_other, consent, EndTime, interviewer_code, supervisor_code, rural, type, oth_interv, numP2, num_teacher, treattype, treatment, treatdummy, endline_dummy	DateofInterview, Starttime, school_code, LGA_code, consent, Endtime, interviewer_code, supervisor_code, rural, type, oth_interv, numP2, num_teacher, treattype, treatment, treatdummy, endline_dummy, SBMC_interviewed BL	DateofInterview, Starttime, school_code, LGA_code, consent, Endtime, interviewer_code, supervisor_code, rural, type, oth_interv, numP2, num_teacher, treattype, treatment, treatdummy, endline_dummy	DateofInterview, Starttime, school_code, LGA_code, consent, Endtime, interviewer_code, supervisor_code, rural, type, oth_interv, numP2, num_teacher, treattype, treatment, treatdummy	DateofInterview, Starttime, school_code, LGA_code, consent, Endtime, interviewer_code, supervisor_code, rural, type, oth_interv, numP2, num_teacher, treattype, treatment, treatdummy, endline_dummy	DateofInterview, Start, school_code, LGA_code, No_Of_Class, LG01*, LG04*, Snap*_OBS00A, Snap*_OBS00B, Snap*_OBS15, Endtime, interviewer_code, supervisor_code, rural, type, oth_interv, numP2, num_teacher, treattype, treatment, treatdummy	DateofInterview, Starttime, school_code, LGA_code, No_Of_Class, LG01*, LG04*, Endtime, interviewer_code, supervisor_code, rural, type, oth_interv, numP2, num_teacher, treattype, treatment, treatdummy, endline_dummy
